# In Vitro Activity and Clinical Outcomes of Clofazimine for Nontuberculous Mycobacteria Pulmonary Disease

**DOI:** 10.3390/jcm10194581

**Published:** 2021-10-03

**Authors:** Dae Hun Kim, Bo-Guen Kim, Su-Young Kim, Hee Jae Huh, Nam Yong Lee, Won-Jung Koh, Hojoong Kim, O Jung Kwon, Byung Woo Jhun

**Affiliations:** 1 Samsung Medical Center, Department of Medicine, Division of Pulmonary and Critical Care Medicine, Sungkyunkwan University School of Medicine, Seoul 06351, Korea; tnxo515@gmail.com (D.H.K.); kbg1q2w3e@gmail.com (B.-G.K.); suyoung5505@gmail.com (S.-Y.K.); healthylung@gmail.com (W.-J.K.); hjk3425@skku.edu (H.K.); ojkwon@skku.edu (O.J.K.); 2 Samsung Medical Center, Department of Laboratory Medicine and Genetics, Sungkyunkwan University School of Medicine, Seoul 06351, Korea; pmhhj77@gmail.com (H.J.H.); micro.lee@samsung.com (N.Y.L.)

**Keywords:** nontuberculous mycobacteria, clofazimine, in vitro, minimum inhibitory concentration, minimum bactericidal concentration

## Abstract

Limited data are available regarding the in vitro activity of clofazimine against nontuberculous mycobacteria (NTM) or on outcomes of clofazimine-containing regimens in NTM-pulmonary disease (PD). Therefore, we evaluated the in vitro activity of clofazimine and the clinical outcomes of clofazimine-containing regimens. We evaluated clofazimine in vitro activity for 303 NTM isolates from NTM-PD patients. Fifty-seven clarithromycin-resistant and 35 amikacin-resistant isolates were also analyzed. Culture conversion after a 12-month treatment regimen containing clofazimine was evaluated in 58 NTM-PD patients, including 20 patients with drug-resistant isolates. Most of the 303 isolates (238/303) had minimum inhibitory concentrations (MICs) ≤ 0.25 µg/mL for clofazimine (57/63 *Mycobacterium avium*, 53/57 *M. intracellulare*, 49/52 *M. kansasii*, 22/64 *M. abscessus*, and 57/67 *M. massiliense*). For the 57 clarithromycin-resistant and 35 amikacin-resistant isolates, most had MICs ≤ 0.25 µg/mL (47/57 and 32/35, respectively). Among the 38 NTM-PD patients without resistance to clarithromycin or amikacin, 47% achieved culture conversion (8/27 *M. abscessus*, 9/9 *M. massiliense*, 0/1 *M. avium*, and 1/1 *M. intracellulare*). The conversion rate was higher in the MIC ≤ 0.25 µg/mL group than in the MIC = 0.5 µg/mL group (13/18 vs. 5/20, *p* = 0.004), and an MIC ≤ 0.25 µg/mL remained a significant factor in multivariable analysis. Culture conversion was achieved in 20% of 20 patients with clarithromycin- or amikacin-resistant isolates. However, a clofazimine MIC ≤ 0.25 µg/mL was not significant for culture conversion in the 58 NTM-PD patients, regardless of the drug resistance pattern. Clofazimine was effective in vitro against NTM species. Some patients on clofazimine-containing regimens achieved culture conversion.

## 1. Introduction

The burdens of pulmonary disease (PD) due to nontuberculous mycobacteria (NTM) are increasing globally [1,2]. *Mycobacterium avium* complex (MAC), mainly composed of *M. avium* and *M. intracellulare*, is the most common pathogen associated with NTM-PD, and *M. abscessus*, predominantly composed of *M. abscessus* subsp. *abscessus* (hereafter, *M. abscessus*) and *M. abscessus* subsp. *massiliense* (hereafter, *M. massiliense*), is the second most common pathogen in many countries. *M. kansasii* also causes NTM-PD [3,4].

For MAC-PD or *M. kansasii*-PD, guidelines recommend a macrolide-based multidrug therapy, including ethambutol and rifamycin with or without an injectable aminoglycoside [4,5]. However, treatment outcomes are unsatisfactory, and many patients remain culture-positive with refractory NTM-PD [6,7]. For *M. abscessus*- or *M. massiliense*-PD, a major challenge is that the pathogens are highly drug-resistant, despite guidelines recommending multidrug therapy, including intravenous amikacin with imipenem (or cefoxitin), based on the results of drug susceptibility testing (DST) [8]. Thus, there are only a few effective oral drugs for long-term maintenance therapy of NTM-PD in real-world practice, and improved therapeutic options are needed.

There is growing evidence on the efficacy of clofazimine for treatment of primary or refractory NTM-PD [9,10,11,12,13]. Clofazimine is a fat-soluble riminophenazine-based antibiotic primarily used to treat leprosy due to its lipophilicity in the skin and anti-inflammatory properties [14], and it has been used to treat drug-resistant tuberculosis [13,15,16]. Clofazimine has several advantages for NTM-PD treatment, including its long half-life, oral availability, slow metabolic elimination, ability to achieve high concentration in macrophages, and rapid localization within phagocytes [17]. Additionally, data have demonstrated synergistic effects with amikacin against NTM [18].

However, limited research exists on the in vitro antibiotic susceptibility for clinical NTM isolates. Therefore, we evaluated the in vitro activity of clofazimine against NTM, including macrolide- or amikacin-resistant clinical isolates, and clinical outcomes of NTM-PD patients treated with clofazimine-containing regimens.

## 2. Materials and Methods

### 2.1. Study Participants

We evaluated 303 clinical isolates of major pathogenic NTMs obtained from 303 patients with treatment-naive NTM-PD between January 2009 and November 2018 at Samsung Medical Center, a referral hospital in Seoul, Korea. Additionally, 57 clarithromycin-resistant and 35 amikacin-resistant non-duplicated NTM isolates, all confirmed to have mutations associated with acquired resistance (*rrl* for clarithromycin and *rrs* for amikacin), were included in the analysis. In vitro activity of clofazimine against these isolates was evaluated. The clinical outcome was also evaluated for 58 patients treated with clofazimine-containing regimens for ≥6 months. All patients met the diagnostic criteria of the American Thoracic Society/Infectious Diseases Society of America guidelines [4]. All NTM isolates and the clinical data included in this study were obtained from an Institutional Review Board-approved observational cohort at Samsung Medical Center (ClinicalTrials.gov identifier: NCT00970801).

### 2.2. NTM Identification

Acid-fast bacilli smears and cultures of the respiratory specimens were obtained using standard methods. The processed specimens were inoculated into the BACTEC MGIT system (BD Diagnostics, Sparks, MD, USA). Liquid cultures were used for NTM species identification and DST. Species were identified using polymerase chain reaction (PCR)-restriction fragment length polymorphism analysis or reverse-blot hybridization of the *rpoB* gene in routine clinical practice. Beginning in June 2014, species identification was conducted via nested multiplex PCR and a reverse-hybridization assay of the internal transcribed spacer (ITS) region (AdvanSureTM Mycobacteria GenoBlot Assay; LG Life Sciences, Seoul, Korea).

For research, NTM isolates from broth medium were subcultured on 3% Ogawa agar before harvesting and storage at −80 °C. Clinical isolates were then propagated on Middlebrook 7H10 agar plates (Difco Laboratories, Detroit, MI, USA) supplemented with 10% (*v*/*v*) oleic acid–albumin–dextrose–catalase (OADC) (BD Diagnostics). Single colonies were obtained and re-identified using multilocus sequence analysis of *hsp65*, the 16S-23S rRNA gene ITS, *erm*(41), and *rpoB* and were retested for clofazimine susceptibility.

### 2.3. In Vitro Activity against Clofazimine

In vitro susceptibility testing of various antibiotics was performed using the broth microdilution method according to the Clinical and Laboratory Standards Institute (CLSI) guidelines [19,20]. Rapidly growing mycobacteria (RGM), including *M. abscessus* and *M. massiliense*, used cation-adjusted Mueller–Hinton broth (CAMHB) (Difco Laboratories), and slowly growing mycobacteria (SGM), including MAC and *M. kansasii*, used CAMHB with 5% OADC according to CLSI guidelines.

Clofazimine dissolved in dimethyl sulfoxide was dispensed into CAMHB or CAMHB with 5% OADC using serial dilution, and then the antibiotic-containing medium was dispensed into 96-well plates. A single colony of bacteria was placed in a 5 mL tube containing phosphate-buffered saline, and the density was adjusted to 0.5 McFarland standard. The bacterial suspension was diluted 1/200, inoculated into CAMHB or CAMHB with 5% OADC, and then dispensed into the 96-well plates.

Minimum inhibitory concentrations (MICs) were determined after incubation of RGM (3–5 days) and SGM (>10 days) at a constant temperature (36 °C) and humidity (75–80%). After MIC measurement, inoculum suspensions (20–50 μL) in 96-well plates at concentrations above MIC were inoculated on Middlebrook 7H10 agar with 10% OADC without antibiotics followed by re-incubation (RGM, 3–4 days; SGM, ≥10 days). Minimum bactericidal concentrations (MBCs) were determined as the lowest antibiotic concentration that yielded no visible colonies.

### 2.4. Sequence Analysis for Acquired Clarithromycin and Amikacin Resistance Mutations

Isolates were categorised as clarithromycin or amikacin resistant, respectively, if they contained any of the following *rrl* or *rrs* mutations associated with acquired resistance [21]: A2058G, A2058C, A2058T, A2059T, A2059C, or A2059G (*Escherichia coli* 23S rRNA gene position numbering) in the clarithromycin acquired resistance region of *rrl*; or A1408G, T1406A, C1409T, or G1491T in the amikacin acquired resistance region of *rrs* (*E. coli* 16S rRNA gene position numbering). To confirm *rrl* and *rrs* gene mutations, NTM DNA was extracted using the DNeasy UltraClean Microbial Kit (Qiagen, Hilden, Germany), and PCR was performed using *rrl* (*rrl*-F; 5′-TTTAAGCCCCAGTAAACGGC-3′; *rrl*-R, 5′-GTCCAGGTTGAGGGAACCTT-3′) or *rrs* (*rrs*1-F; 5′-ATGACGTCAAGTCAT CATGCC-3′; *rrs*1-R, 5′-AGGTGATCCAGCCGCACCTTC-3′) specific primers, respectively. The following PCR conditions were used for the *rrl* and *rrs* regions: denaturation at 94 °C (2 min) followed by 35 cycles (94 °C, 30 s; 58.5 °C, 30 s; 72 °C, 3 min) and a final extension for 5 min. The PCR products were purified and sequenced using the *rrl*-F and *rrs*1-F primers, respectively.

### 2.5. Treatment and Clinical Outcomes

We evaluated treatment outcomes for 58 patients with NTM-PD (*M. abscessus*, *M. massiliense*, or MAC) who were treated with clofazimine-containing regimens for ≥6 months. In *M. abscessus-* or *M. massiliense*-PD patients, an initial two- to four-week course of intravenous amikacin and imipenem (or cefoxitin) with or without tigecycline was administered, together with oral macrolide or clofazimine (100 mg/day), after which the oral regimen was maintained. In MAC-PD patients, a macrolide-based regimen including ethambutol and rifampin, with or without intravenous amikacin, was used. Non-liposomal inhaled amikacin was used for patients with refractory NTM-PD (Supplementary Table S1). At our institution, clofazimine was included in the initial *M. abscessus* or *M. massiliense* treatment, while clofazimine was usually added as salvage treatment in refractory MAC-PD.

Treatment outcome was based on culture conversion at 12 months after antibiotic treatment initiation. ‘Culture conversion’ was defined as at least three consecutive negative cultures after treatment, collected at least four weeks apart. Time of conversion was defined as the date of the first negative culture. There are two reasons for evaluating the outcomes at 12 months after treatment initiation: first, treatment outcomes could be underestimated in patients with shorter treatment periods and overestimated in those with longer treatment periods; second, in real-world clinical practice, treatment outcome is evaluated after 12 months of therapy.

### 2.6. Statistical Analysis

Data were presented as numbers (percentages) or medians (interquartile ranges). Data were compared by the Mann–Whitney U test for continuous variables and the chi-square test or Fisher’s exact test for categorical variables. Multivariable analyses with a logistic regression model were performed to evaluate factors associated with culture conversion. Differences were considered statistically significant at *p* < 0.05. All statistical analyses were performed using SPSS (IBM SPSS Statistics ver. 25, Chicago, IL, USA).

## 3. Results

### 3.1. In Vitro Activity of Clofazimine in NTM without Drug Resistance

Table 1 shows the MIC and MBC values of clofazimine against 303 clinical NTM isolates without resistance to clarithromycin and amikacin. Overall, the MICs of clofazimine tended to be low in all NTM species. Most of the isolates, 79% (238/303), had clofazimine MICs ≤ 0.25 µg/mL, including 90% (57/63) of *M. avium*, 93% (53/57) of *M. intracellulare*, 94% (49/52) of *M. kansasii*, 34% (22/64) of *M. abscessus*, and 85% (57/67) of *M. massiliense*.

The MIC of clofazimine for MAC or *M. kansasii* tended to be lower than that of *M. abscessus* or *M. massiliense*. The MIC_50_ and MIC_90_ of clofazimine against *M. kansasii* were ≤0.062 µg/mL and 0.125 µg/mL, respectively, showing the lowest values among all NTM species. The MIC_50_ and MIC_90_ of clofazimine for *M. abscessus* were the highest at 0.5 µg/mL. The MBC values of clofazimine in each NTM species showed similar trends to the MIC values.

### 3.2. In Vitro Activity of Clofazimine in NTM with Drug Resistance

Clofazimine MICs and MBCs were also determined against 57 clarithromycin- and 35 amikacin-resistant NTM (Table 2), which were identified through sequence analysis for mutations associated with acquired resistance to clarithromycin and amikacin, respectively. Overall, the clofazimine MICs were low in all NTM species regardless of resistance to clarithromycin or amikacin. For clarithromycin-resistant isolates, most of the isolates, 82% (47/57), had clofazimine MICs ≤ 0.25 µg/mL, including all (10/10) of *M. avium*, 88% (15/17) of *M. intracellulare*, 62% (8/13) of *M. abscessus*, and 82% (14/17) of *M. massiliense*. The MIC_50_ and MIC_90_ values of clofazimine were 0.25 µg/mL and 0.5 µg/mL in *M. abscessus* and *M. massiliense*, respectively, higher than those in *M. avium* or *M. intracellulare*.

For amikacin-resistant isolates, all *M. avium* isolates showed the lowest clofazimine MICs, ≤0.062 µg/mL. Clofazimine MICs of ≤0.25 µg/mL were found for 93% (14/15) of *M. intracellulare*, 83% (5/6) of *M. abscessus*, and 67% (2/3) of *M. massiliense* isolates. MIC_50_ and MIC_90_ values in *M. abscessus* or *M. massiliense* were higher than those in *M. avium* or *M. intracellulare*. Overall, clofazimine MBC and MIC values showed similar trends.

### 3.3. Characteristics of Patients Treated with Clofazimine-Containing Regimens

For the 58 patients treated with clofazimine-containing regimens for ≥6 months, clinical characteristics were evaluated at the regimen start time (Table 3). Of them, 40 patients received clofazimine for ≥12 months, and 18 patients received clofazimine for 6–12 months. The median age was 61 years, and 69% of patients were female. The most common underlying condition was a previous history of pulmonary tuberculosis (50%), followed by chronic pulmonary aspergillosis (10%). The most common etiologic organism was *M. abscessus* (60%), followed by *M. massiliense* (26%). The nodular bronchiectatic form of NTM-PD was present in 37 (64%) patients, with (15/37) or without (22/37) cavity, and the remaining 21 (36%) had the fibrocavitary form. Isolates resistant to clarithromycin or amikacin were confirmed in 20 patients, 14 of whom had *M. abscessus*-PD or *M. massiliense*-PD; the remaining six had *M. avium-*PD or *M. intracellulare*-PD.

### 3.4. Treatment Outcome at 12 Months after Clofazimine-Containing Regimens

Table 4 shows the culture conversion data of 38 NTM-PD patients without drug-resistant isolates. Sputum culture conversion was achieved in 47% (18/38) of patients, including 8/27 of *M. abscessus*, 9/9 of *M. massiliense*, 0/1 of *M. avium*, and 1/1 of *M. intracellulare* (Supplementary Figure S1). Regardless of NTM species, as the MIC decreased, the conversion rate tended to be higher (Supplementary Figure S2A, *p* = 0.004). Also, the conversion rate was significantly higher in patients with MICs ≤ 0.25 µg/mL than with MICs = 0.5 µg/mL (Supplementary Figure S2B, *p* = 0.004). After adjusting for various clinical, laboratory, and demographic factors, clofazimine MICs ≤ 0.25 µg/mL remained a significant factor associated with culture conversion (Table 5).

For the 20 NTM-PD patients with clarithromycin- or amikacin-resistant isolates, culture conversion was achieved in 20% (4/20), including 3/14 of *M. abscessus* and 1/6 of *M. intracellulare* (Supplementary Figure S3). However, the conversion rate according to MIC value was not statistically significant.

Overall, as MIC decreased, the conversion rate among all 58 NTM-PD patients trended higher, but the trend was not statistically significant (Supplementary Figure S4A,B). Furthermore, after adjusting for various factors, MICs ≤ 0.25 µg/mL of clofazimine were not a significant factor associated with culture conversion (Supplementary Table S2).

## 4. Discussion

This study revealed that clofazimine was effective in vitro against major pathogenic NTM species, regardless of resistance to key antibiotics such as clarithromycin or amikacin. In addition, our data showed that lower MICs of clofazimine were associated with higher culture conversion rates at 12 months after treatment in NTM-PD patients, especially for NTM without resistance to key antibiotics. Thus, our data imply that clofazimine could be a viable therapeutic option for patients with NTM-PD.

Our data showed that 80–90% of NTM isolates had clofazimine MICs ≤ 0.25 µg/mL regardless of drug resistance pattern, except for *M. abscessus*, for which 34% of drug-susceptible isolates and 62% of isolates with clarithromycin and/or amikacin resistance had clofazimine MICs ≤ 0.25 µg/mL. Notably, MIC or MBC values in *M. kansasii* were the lowest among all the NTM species in our study. Similar to our present findings, previous studies analyzing in vitro activity of clofazimine have shown a generally low distribution of MIC values across NTM species with various MIC levels [18,22,23,24,25]. In a Taiwanese study evaluating RGM, approximately 99% of 117 *M. abscessus* isolates had clofazimine MICs ≤ 1 µg/mL [22]. Jakko et al. demonstrated MIC_50_ and MIC_90_ of ≤0.5 µg/mL and 1.0 µg/mL, respectively, in a study that evaluated synergy between clofazimine and amikacin using 342 *M. abscessus* isolates [18]. In a MAC study from China, clofazimine demonstrated strong activity with tentative epidemiological cutoffs at 1 µg/mL and 2 µg/mL for MIC_50_ and MIC_90_, respectively [25]. Nevertheless, a more remarkable finding of our study is that, despite clarithromycin or amikacin resistance, most NTM isolates had low clofazimine MIC or MBC values across NTM species. For example, all (10/10) clarithromycin-resistant *M. avium* and 83% (5/6) of amikacin-resistant *M. abscessus* had clofazimine MICs ≤0.25 µg/mL. We also found low MIC values for *M. kansasii*, which has not been reported in previous studies. In this context, given that there are limited data on the in vitro activity of clofazimine using large sample sizes and various types of clinical NTM strains, our results provide important insights.

A major challenge in managing NTM-PD, especially refractory PD, is the lack of sufficient and appropriate active drugs that can be used throughout long-term maintenance therapy. For MAC-PD, although guidelines recommend macrolide-based multidrug therapy including intravenous- or liposomal-inhaled amikacin based on the results of DST [4,5,26], no effective oral agent has been shown other than rifampin and ethambutol. Especially for *M. abscessus*-PD, the pathogen is highly drug-resistant, and long-term use of injectable antibiotics is not feasible in real-world practice. The efficacy of liposomal-inhaled amikacin in treatment of MAC-PD has not been proven in *M. abscessus*-PD. Moreover, only clarithromycin and amikacin have demonstrated a correlation between clinical outcome and in vitro activity. Thus, there is a need for research on drugs that can be selected when guideline-recommended drugs become unavailable or ineffective due to adverse effects or resistance. Given this context, our results suggest that clofazimine could be considered, depending on MIC level, in NTM-PD treatment.

Another important finding of our study was that lower clofazimine MIC values were associated with culture conversion in NTM-PD patients. This phenomenon was more clearly observed in 38 NTM-PD patients without resistance to key antibiotics such as clarithromycin or amikacin. As the MIC decreased, the conversion rate increased and was significantly higher in patients with MICs ≤ 0.25 µg/mL than in those with MIC = 0.5 µg/mL of clofazimine (Supplementary Figure S2). Moreover, an MIC ≤ 0.25 µg/mL remained a significant factor in multivariable analysis (Table 5). However, when adding patients affected by drug-resistant NTM to the above analysis, the association between lower MIC and culture conversion was not significant (Supplementary Figure S4 and Supplementary Table S2). These findings imply that clarithromycin or amikacin susceptibility is critical in treatment of NTM-PD, in all combinations of clofazimine and other agents. Indeed, prior studies have indicated poor treatment outcomes after the occurrence of acquired macrolide resistance in MAC- or *M. abscessus*-PD patients [27,28,29,30]. Previous studies have reported unsatisfactory clinical outcomes of salvage clofazimine treatment in refractory MAC- or *M. abscessus*-PD, with a less than 30% conversion rate [12,13]. Additionally, given the synergism between clofazimine and amikacin (or clarithromycin) [18,24,31,32], it is estimated that this strategy will not be effective in cases of resistance to clarithromycin or amikacin. Thus, more data on the clinical applicability of clofazimine are needed.

Interestingly, in our study, among the 27 patients with *M. abscessus* without acquired drug resistance to clarithromycin or amikacin, culture conversion rates tended to be higher in patient groups with low MICs of clofazimine (43% (3/7) of the MIC = 0.25 µg/mL group vs. 25% (5/20) of the MIC = 0.5 µg/mL group, Supplementary Figure S1). This finding suggests that, when *M. abscessus* shows a low clofazimine MIC without drug resistance against a macrolide or aminoglycoside, clofazimine is worth considering as a therapeutic agent. As noted previously, clofazimine has several advantages for treatment of NTM-PD. However, to date, limited data exist on the pharmacokinetics of clofazimine, and there is no consensus on the optimal dosage or administration interval. Thus, further studies are warranted on its use for NTM-PD.

There are several limitations to our study. First, our MIC data might not be generalizable to other geographic areas. Second, several factors might have influenced the clinical outcome. As the outcome was mainly evaluated after 12 months, the final treatment outcome might have differed if the treatment period was extended. In addition, some patients received clofazimine for less than 12 months, and the number of patients included in the clinical outcome analysis and multivariate analysis was small. Lastly, we did not evaluate the mechanism associated with high MIC of clofazimine in NTM species. Zhang et al. showed that 97% of clofazimine-resistant *M. tuberculosis* strains had mutations in Rv0678 [33]; however, limited data are available on the association between gene mutations and resistance to clofazimine in NTM species. In a recent Chinese study that evaluated in vitro susceptibilities to clofazimine of clinical NTM isolates, there was no specific mutation within the Rv0678 among 22 *M. avium* isolates, and only 2/35 *M. intracellulare* isolates with MICs ≥ 8 μg/mL had Rv0678 mutation [25]. Further studies on these topics are needed.

In conclusion, we found that clofazimine had effective in vitro activity for major pathogenic NTM species regardless of resistance to key antibiotics such as clarithromycin or amikacin. Given that lower MIC values of clofazimine are associated with higher culture conversion rates at 12 months after treatment in NTM-PD patients without drug resistance, clofazimine could be a potential therapeutic option for patients with NTM-PD. However, further research on the effect of clofazimine in patients with isolates resistant to key antibiotics such as clarithromycin and amikacin is necessary.

## Figures and Tables

**Table 1:** MIC_50_, MIC_90_, MBC_50_, and MBC_90_ values of clofazimine for 303 clinical NTM isolates.

NTM Species (No.)	Activity	No. (%) of Isolates According to MIC (µg/mL) *							MIC_50_ (µg/mL)	MIC_90_ (µg/mL)	MBC_50_ (µg/mL)	MBC_90_ (µg/mL)
		≤0.062	0.125	0.25	0.5	1	2	4				
*M. avium* (63)	MIC	23 (36)	16 (25)	18 (29)	3 (5)	1 (2)	2 (3)		0.125	0.25		
	MBC	20 (31)	15 (24)	20 (32)	5 (8)	1 (2)	2 (3)				0.125	0.5
*M. intracellulare* (57)	MIC	5 (8)	20 (35)	28 (49)	2 (4)		2 (4)		0.25	0.25		
	MBC	5 (8)	15 (27)	32 (56)	3 (5)		2 (4)				0.25	0.25
*M. kansasii* (52)	MIC	45 (86)	3 (6)	1 (2)	1 (2)		2 (4)		≤0.062	0.125		
	MBC	39 (75)	9 (17)	1 (2)		1 (2)	1 (2)	1 (2)			≤0.062	0.125
*M. abscessus* (64)	MIC		2 (3)	20 (31)	42 (66)				0.5	0.5		
	MBC			2 (3)	58 (91)	4 (6)					0.5	0.5
*M. massiliense* (67)	MIC	2 (3)	4 (6)	51 (76)	10 (15)				0.25	0.5		
	MBC	1 (2)		25 (37)	41 (61)						0.5	0.5

* Data are presented as number (%). MIC: minimum inhibitory concentration; MBC: minimum bactericidal concentration; NTM: nontuberculous mycobacteria.

**Table 2:** MIC_50_, MIC_90_, MBC_50_, and MBC_90_ values of clofazimine for 57 clarithromycin-resistant and 35 amikacin-resistant NTM isolates *.

NTM Species (No.)	Activity	No. (%) of Isolates According to MIC (µg/mL) *					MIC_50_ (µg/mL)	MIC_90_ (µg/mL)	MBC_50_ (µg/mL)	MBC_90_ (µg/mL)
		≤0.062	0.125	0.25	0.5	1				
***Clarithromycin-resistant***										
*M. avium* (10)	MIC	4 (40)	6 (60)				0.125	0.125		
	MBC	3 (30)	5 (50)	2 (20)					0.125	0.25
*M. intracellulare* (17)	MIC	3 (17)	8 (47)	4 (24)	2 (12)		0.125	0.5		
	MBC	3 (17)	5 (30)	5 (30)	3 (17)	1 (6)			0.25	0.5
*M. abscessus* (13)	MIC		2 (15)	6 (46)	4 (31)	1 (8)	0.25	0.5		
	MBC		1 (8)	4 (31)	7 (53)	1 (8)			0.5	0.5
*M. massiliense* (17)	MIC		6 (35)	8 (47)	3 (18)		0.25	0.5		
	MBC			7 (42)	10 (58)				0.5	0.5
***Amikacin-resistant***										
*M. avium* (11)	MIC	11 (100)					≤0.062	≤0.062		
	MBC	11 (100)							≤0.062	≤0.062
*M. intracellulare* (15)	MIC	9 (60)	3 (20)	2 (13)	1 (7)		≤0.062	0.25		
	MBC	7 (46)	4 (27)	3 (20)	1 (7)				0.125	0.25
*M. abscessus* (6)	MIC			5 (83)	1 (17)		0.25	0.5		
	MBC				6 (100)				0.5	0.5
*M. massiliense* (3)	MIC			2 (67)	1 (33)		0.5	0.5		
	MBC			1 (33)	2 (67)				0.5	0.5

* Data are presented as number (%). MIC: minimum inhibitory concentration; MBC: minimum bactericidal concentration; NTM: nontuberculous mycobacteria.

**Table 3:** Characteristics of patients treated with clofazimine-containing regimens (*n* = 58).

Characteristics	Values
Age, years	61 (55–69)
Sex, female	40 (69)
Body mass index, kg/m^2^	20.9 (18.1–22.2)
Never-smoker	43 (74)
Comorbidity	
Previous history of pulmonary tuberculosis	29 (50)
Chronic pulmonary aspergillosis	6 (10)
Obstructive pulmonary disease	5 (9)
Diabetes mellitus	5 (9)
Chronic heart disease	4 (7)
Chronic liver disease	2 (3)
Previous lung cancer	1 (2)
Other malignancy *	2 (3)
Etiologic organism	
*M. abscessus*	35 (60)
*M. massiliense*	15 (26)
*M. avium*	3 (5)
*M. intracellulare*	5 (9)
Radiologic findings	
Nodular bronchiectatic form	37 (64)
With cavity	15/37
Without cavity	22/37
Fibrocavitary form	21 (36)
Laboratory findings	
Positive sputum acid-fast bacilli smear	41 (71)
Erythrocyte sedimentation rate, mm/h	55.0 (24.3–68.3)
Clarithromycin or amikacin resistance ^¶^	20 (34)
*M. abscessus* or *M. massiliense*	14/20
*M. avium* or *M. intracellulare*	6/20

Data are presented as number (%) or median (interquartile range). * Rectal cancer (*n* = 1), sarcoma (*n* = 1). ^¶^ Isolates resistant to macrolide or aminoglycoside or both.

**Table 4:** Culture conversion rate after 12 months of treatment in patients without drug-resistant isolates according to MIC value and NTM species (*n* = 38).

	No Conversion					Conversion					
MIC	*M. abscessus*	*M. massiliense*	*M. avium*	*M. intracellulare*	Total *	*M. abscessus*	*M. massiliense*	*M. avium*	*M. intracellulare*	Total ^¶^	*p*-Value
≤0.062	-	-	-	-	-	-	1	-	-	1	0.004
0.125	-	-	1	-	1	-	-	-	-	-	
0.25	4	-	-	-	4	3	8	-	1	12	
0.5	15	-	-	-	15	5	-	-	-	5	
MIC	*M. abscessus*	*M. massiliense*	*M. avium*	*M. intracellulare*	Total *	*M. abscessus*	*M. massiliense*	*M. avium*	*M. intracellulare*	Total ^¶^	*p*-value
≤0.25	4	-	1	-	5	3	9	-	1	13	0.004
≥0.5	15	-	-	-	15	5	-	-	-	5	

Data are presented as number. *p*-value is the value comparing Total * and Total ^¶^ regardless of NTM species. MIC: minimum inhibitory concentration; NTM: nontuberculous mycobacteria.

**Table 5:** Factors associated with culture conversion at 12 months in patients without drug-resistant isolates (*n* = 38).

Variable	Univariable		Multivariable	
	Unadjusted HR (95% CI)	*p* Value	Adjusted HR (95% CI)	*p* Value
Age < 65 years	1.500 (0.534–4.214)	0.442	3.099 (0.680–14.129)	0.144
Female	0.626 (0.206–1.909)	0.411	-	-
Body mass index ≥ 18.5 kg/m^2^	1.496 (0.561–3.990)	0.421	1.452 (0.374–5.631)	0.590
Never smoker	1.347 (0.442–4.102)	0.600	-	-
No previous pulmonary tuberculosis	0.825 (0.326–2.092)	0.686	0.953 (0.232–3.906)	0.947
Etiology				
*M. abscessus*	Reference		Reference	
*M. massiliense*	9.179 (3.116–27.040)	<0.001	51.978 (1.463–1846.164)	0.030
*M. avium*/*M. intracellulare*	1.373 (0.172–10.987)	0.765	0.214 (0.004–11.608)	0.449
Negative sputum acid-fast bacilli smear	1.738 (0.681–4.431)	0.247	1.521 (0.416–5.564)	0.526
No cavity	0.877 (0.329–2.339)	0.793	130.124 (4.423–3827.900)	0.005
MIC of clofazimine, µg/mL				
≥0.5	reference		reference	
≤0.25	4.382 (1.548–12.408)	0.005	22.458 (1.595–316.251)	0.021
Macrolide resistance				
Inducible resistance	Reference		Reference	
Susceptible	3.452 (1.325–8.995)	0.011	0.274 (0.018–4.105)	0.349
Intravenous amikacin use, days	0.974 (0.944–1.005)	0.095	0.956 (0.900–1.016)	0.149
Elevated erythrocyte sedimentation rate *	0.292 (0.039–2.195)	0.232	0.754 (0.067–8.424)	0.818

HR: hazard ratio; CI: confidential interval; MIC: minimum inhibitory concentration. * Erythrocyte sedimentation rate >15 mm/h (men) or >20 mm/h (women).

## Supplementary Materials

The following are available online at www.mdpi.com/article/10.3390/jcm10194581/s1, Figure S1: Culture conversion rate after 12 months of treatment in NTM-PD patients without drug-resistant isolates according to MIC value of clofazimine and NTM species (*n* = 38), Figure S2: (A) Culture conversion rate after 12 months of treatment in NTM-PD patients without drug-resistant isolates according to MIC value of clofazimine, regardless of NTM species (*n* = 38), (B) Culture conversion rate after 12 months of treatment in NTM-PD patients without drug-resistant isolates according to clofazimine MIC of 0.25 µg/mL, regardless of NTM species (*n* = 38), Figure S3: Culture conversion rate after 12 months of treatment in NTM-PD patients with drug-resistant isolates (*n* = 20), Figure S4: (A) Culture conversion rate after 12 months of treatment in all study patients according to MIC value of clofazimine, regardless of NTM species (*n* = 58), (B) Culture conversion rate after 12 months of treatment in all study patients according to clofazimine MIC of 0.25 µg/mL, regardless of NTM species (*n* = 58), Table S1: Companion drugs used with clofazimine, Table S2: Factors associated with culture conversion in patients treated with clofazimine-containing regimens (*n* = 58).

## Abbreviations

PD	pulmonary disease
NTM	nontuberculous mycobacteria
MAC	mycobacterium avium complex
DST	drug susceptibility testing
CLSI	Clinical and Laboratory Standards Institute
RGM	rapidly growing mycobacteria
SGM	slowly growing mycobacteria
MIC	minimum inhibitory concentration
MBC	minimum bactericidal concentration
