# Supplementary material for: In Vitro Activity and Clinical Outcomes of Clofazimine for Nontuberculous Mycobacteria Pulmonary Disease
Source: J Clin Med. 2021 Oct 3;10(19):4581. doi: 10.3390/jcm10194581 (PMC8509216; doi:10.3390/jcm10194581)
Supplement: Supplementary file 1 [file jcm-10-04581-s001.zip › jcm-1369845supplementary table.pdf]

[Supplement data]

In vitro activity and clinical outcomes of clofazimine for nontuberculous mycobacteria pulmonary disease

Dae Hun Kim[1,*], Bo-Guen Kim[1,*], Su-Young Kim[1], Hee Jae Huh[2], Nam Yong Lee[2], Won-Jung Koh[1,†], Hojoong Kim[1], O Jung Kwon[1], Byung Woo Jhun[1]

[1]Division of Pulmonary and Critical Care Medicine, Department of Medicine, Samsung Medical Center, Sungkyunkwan University School of Medicine, Seoul, South Korea. [2]Department of Laboratory Medicine and Genetics, Samsung Medical Center, Sungkyunkwan University School of Medicine, Seoul, South Korea.

[*]These authors contributed equally to this work

†Deceased

Supplementary Table S1 Companion drugs used with clofazimine

Variables	Number (%)
M. abscessus (n=27)	
Companion drugs used with clofazimine	
Azithromycin	27 (100)
Amikacin injection	23 (85)
Inhaled amikacin	23 (85)
Imipenem	21 (78)
Tigecycline	9 (33)
Linezolid	7 (26)
Cefoxitin	6 (22)
Ethambutol	2 (7)
Rifamycin	2 (7)
M. massiliense (n=9)	
Companion drugs used with clofazimine	
Azithromycin	9 (100)
Inhaled amikacin	9 (100)
Amikacin injection	6 (67)
Imipenem	4 (44)
Cefoxitin	1 (11)
M. avium (n=1)	
Azithromycin	1 (100)
Ethambutol	1 (100)
Moxifloxacin	1 (100)
M. intracellulare (n=1)	
Azithromycin	1 (100)
Ethambutol	1 (100)
Inhaled amikacin	1 (100)
Drug-resistant *M. abscessus* or *M. massiliense* (n=14)¶	
Azithromycin	14 (100)
Inhaled amikacin	10 (71)
Amikacin injection	9 (64)
Imipenem	7 (50)
Linezolid	7 (50)
Cefoxitin	5 (36)
Tigecycline	2 (14)
Drug-resistant *M. intracellulare* or *M. avium* (n = 6)¶	
Ethambutol	6 (100)
Azithromycin	5 (83)
Inhaled amikacin	5 (83)
Rifamycin	3 (50)
Linezolid	2 (33)

Amikacin injection	1 (17)
Cefoxitin	1 (17)
Imipenem	1 (17)
Tigecycline	1 (17)
Isoniazid	1 (17)
Moxifloxacin	1 (17)

¶Strains resistant to macrolide or aminoglycoside or both.

Supplementary Table S2 Factors associated with culture conversion in patients treated with clofazimine-containing regimens (n=58)

Variable	Univariable		Multivariable	
	Unadjusted HR (95% CI)	*p* value	Adjusted HR (95% CI)	*p* value
Age <65 years	1.510 (0.590-3.863)	0.390	1.129 (0.381-3.345)	0.826
Female	0.431 (0.146-1.275)	0.128	-	-
Body mass index ≥18.5 kg/m^2	0.818 (0.334-2.008)	0.662	0.869 (0.275-2.750)	0.812
Never smoker	1.706 (0.577-5.044)	0.334	-	-
No previous pulmonary tuberculosis	1.310 (0.566-3.032)	0.529	1.626 (0.530-4.993)	0.396
Etiology				
M. abscessus	Reference		Reference	
M. massiliense	3.143 (1.297-7.618)	0.011	2.130 (0.439-10.342)	0.348
MAC	0.846 (0.185-3.864)	0.830	0.221 (0.022-2.215)	0.199
Negative sputum acid-fast bacilli smear	2.007 (0.0855-4.712)	0.110	1.103 (0.388-3.140)	0.854
**No cavity**	**0.977 (0.410-2.331)**	**0.958**	**4.273 (1.009-18.089)**	**0.049**
MIC of clofazimine, μg/ml				
≥0.5	Reference		Reference	
≤0.25	2.001 (0.815-4.914)	0.130	2.545 (0.587-11.031)	0.212
Macrolide resistance				
Resistant	Reference		Reference	
Inducible resistance	1.665 (0.430-6.440)	0.460	2.775 (0.332-23.160)	0.346
**Susceptible**	**4.582 (1.287-16.312)**	**0.019**	**7.515 (1.164-48.512)**	**0.034**
Intravenous amikacin use, days	0.983 (0.958-1.009)	0.195	0.874 (0.938-1.011)	0.163

Elevated erythrocyte sedimentation rate*	0.481 (0.112-2.061)	0.324	0.896 (0.173-4.638)	0.896

HR, hazard ratio; CI, confidential interval; MAC, *Mycobacterium avium* complex; MIC, minimum inhibitory concentration.

*Erythrocyte sedimentation rate >15 mm/h (men) or >20 mm/h (women).
